# Estratégia Farmacoinvasiva no Infarto do Miocárdio com Supradesnivelamento do Segmento ST: Particularidades no Idoso

**DOI:** 10.36660/abc.20220885

**Published:** 2023-01-31

**Authors:** Fernando Cesena

**Affiliations:** 1 Cenocor Guarulhos SP Brasil Cenocor, Guarulhos, SP – Brasil

**Keywords:** Idoso, Infarto do Miocárdio com Supradesnivelamento do Segmento ST, Intervenção Coronária Percutânea, Terapia Trombolítica/métodos, Revascularização do Miocárdio

O manejo do infarto do miocárdio (IM) em idosos é diferente do tratamento de indivíduos mais jovens. Nos idosos, a apresentação do IM é frequentemente atípica, mais complexa e tem um pior prognóstico. Não só a gravidade da doença e a prevalência de comorbidades tendem a ser maiores nos idosos, como também são mais frequentes os eventos adversos dos tratamentos, principalmente os sangramentos facilitados por diferentes medicações antitrombóticas. Além disso, a diminuição da função renal e a maior suscetibilidade a interações medicamentosas devido ao uso concomitante de múltiplos medicamentos potencializam a chance de desfechos ruins.

No IM com supradesnivelamento do segmento ST (IAMCST), a fibrinólise seguida pela intervenção coronária percutânea (ICP) precoce para otimizar a reperfusão coronária, a chamada estratégia farmacoinvasiva, é uma terapia recomendada por diretrizes quando a ICP não está prontamente disponível.^1 - 4^

Os Arquivos Brasileiros de Cardiologia publicaram um artigo comparando indivíduos <65 anos versus 65-75 anos submetidos à fibrinólise seguida de coronariografia invasiva em até 24 horas após IAMCSST no Brasil.^5^ Depois de excluir pacientes que morreram, foram readmitidos no hospital, encaminhados para cirurgia de revascularização do miocárdio ou tiveram insuficiência renal, os autores avaliaram 223 participantes com ressonância magnética cardíaca (RM, 30 dias após o IM) e um conjunto abrangente de marcadores inflamatórios (nos dias 1 e 30 após o IM). Os autores observaram pequenas diferenças nos parâmetros inflamatórios entre os grupos que não pareceram afetar os resultados da RM. Em comparação com o grupo mais jovem, os participantes mais idosos tiveram uma massa infartada semelhante e fração de ejeção do ventrículo esquerdo levemente maior um mês após o IM. O estudo não teve o objetivo de avaliar desfechos clínicos duros, mas os resultados alinham-se ao conceito atual de que a terapia farmacoinvasiva deve ser realizada quando esteja apropriadamente indicada, independentemente da idade, pelo menos até os 75 anos.

A pergunta mais relevante é: e os indivíduos com mais de 75 anos? A esse respeito, algumas observações podem ser feitas. Primeiro, as preocupações sobre o risco de hemorragia que ameace a vida após a fibrinólise em idosos são justificáveis. A idade avançada é um preditor claro de sangramento importante após a fibrinólise. No estudo STREAM, uma taxa excessiva de hemorragia intracraniana (HIC) em participantes ≥75 anos levou a uma alteração do protocolo recomendando uma redução de 50% na dose de tenecteplase nessa faixa etária.^1^ Uma extensa análise de pacientes tratados com dose total de tenecteplase ou alteplase mostrou que o risco de sangramento maior ou HIC começa a aumentar a partir dos 60 anos.^6^

Em segundo lugar, é amplamente aceito que os benefícios da reperfusão coronariana com trombólise superam o risco de sangramento mesmo naqueles indivíduos >75 anos. De fato, a idade avançada não é nem mesmo uma contra-indicação relativa para fibrinólise em IAMCST.^3 , 4^ Na fase pós-alteração do protocolo do estudo STREAM, não houve HIC com meia dose de tenecteplase e a eficácia da estratégia farmacoinvasiva foi mantida em 93 idosos participantes.^7^

Em terceiro lugar, os médicos devem prestar atenção às particularidades do tratamento farmacoinvasivo em pacientes idosos com IAMCST. Não só a dose de tenecteplase deve ser cortada pela metade em indivíduos ≥75 anos, mas a dose de ataque de 300 mg de clopidogrel e o bolus intravenoso inicial de 30 mg de enoxaparina também devem ser omitidos. Uma redução de 25% na dose de enoxaparina também é recomendada, pelo menos na fase inicial do tratamento. Além disso, na presença de disfunção renal, deve-se ajustar a dose de enoxaparina ou usar heparina não fracionada.^3 , 4^ Erros de dosagem de antitrombóticos em idosos com IM podem ser comuns e impõem um risco mais alto de sangramento maior.^8^

Em quarto lugar, o regime antitrombótico ideal em indivíduos idosos no contexto de uma estratégia farmacoinvasiva em IAMCST é uma questão em evolução, e as recomendações podem mudar à medida que novas evidências surgirem. Por exemplo, o estudo STREAM-2 em andamento avalia a eficácia e a segurança de meia dose de tenecteplase e terapia antiplaquetária, incluindo uma dose de ataque de 300 mg de clopidogrel, em comparação com a ICP primária padrão, em pacientes com IAMCST com idade ≥60 anos.^6^

A discussão sobre a segurança da estratégia farmacoinvasiva deve ser inserida no contexto mais amplo da qualidade da assistência ao IAMCSST. No Brasil, há espaço para melhorias em vários aspectos do tratamento do IAMCSST, incluindo as taxas de terapia de reperfusão.^9^ No Registro Brasileiro de Síndromes Coronárias Agudas (ACCEPT, pacientes incluídos de 2010 a 2014), quase 20% dos participantes não receberam terapia de reperfusão para IAMCSST, e essa taxa sobe para ~35% nas Regiões Centro-Oeste e Norte.^10^ A chegada tardia ao pronto-socorro e as contraindicações para fibrinólise podem ser responsáveis por vários casos. No entanto, alguns indivíduos mais idosos podem não receber medicamentos fibrinolíticos devido a preocupações com o risco de sangramento, o que não é suportado pela literatura. Em conclusão, os indivíduos mais idosos constituem um grupo especial de pacientes com IAMCST. Estudos abordando as particularidades do tratamento nessa população são bem-vindos e necessários para fornecer evidências adequadas para otimizar o atendimento médico.
