# Supplementary material for: Estratégia Farmacoinvasiva no Infarto do Miocárdio com Supradesnivelamento do Segmento ST: Particularidades no Idoso
Source: Arq Bras Cardiol. 2023 Jan 31;120(1):e20220885. [Article in Portuguese] doi: 10.36660/abc.20220885 (PMC10389105; doi:10.36660/abc.20220885)
Supplement: Translation

# Translation: Pharmacoinvasive Strategy in ST-elevation Myocardial Infarction: Particularities in the Elderly

**Authors:** Fernando Cesena

**Affiliations:** 1 Brazil Cenocor, Guarulhos, SP – Brazil

**Keywords:** Aged, ST Elevation Myocardial Infarction, Percutaneous Coronary Intervention, Thrombolytic Therapy/methods, Myocardial Revascularization

Managing myocardial infarction (MI) in older people differs from treating younger individuals. In the elderly, MI presentation is often atypical, more complex, and has a worse prognosis. Not only do the disease severity and the prevalence of comorbidities tend to be higher in the elderly, but adverse events from treatments are also more frequent, particularly bleeding facilitated by different antithrombotic medications. Moreover, decreased renal function and the higher susceptibility to drug interactions due to the concomitant use of multiple medications potentiate the chance of bad outcomes.

In ST-elevation MI (STEMI), fibrinolysis followed by the early percutaneous coronary intervention (PCI) to optimize coronary reperfusion, the so-called pharmacoinvasive strategy, is a guideline-recommended therapy when timely PCI is not available.^1 - 4^

The *Arquivos Brasileiros de Cardiologia* published an article comparing individuals <65 years versus 65-75 years who underwent fibrinolysis followed by invasive coronary angiography within 24 hours after STEMI in Brazil.^5^After excluding patients who died, were readmitted to the hospital, referred for coronary artery bypass surgery, or had renal failure, the authors evaluated 223 participants with cardiac magnetic resonance (MR, 30 days after the MI) and a comprehensive set of inflammatory markers (at days 1 and 30 after the MI). The authors observed minor differences in inflammatory parameters between the groups that did not seem to impact MR outcomes. Compared to the younger group, older participants had a similar infarcted mass and mildly higher left ventricular ejection fraction one month after the MI. The study did not have the objective of evaluating hard clinical endpoints, but the results align with the current concept that pharmacoinvasive therapy should be performed when properly indicated, regardless of age, at least until 75 years.

The more relevant question is: what about individuals older than 75 years? In this regard, some remarks can be made. First, concerns about the risk of life-threatening hemorrhage after fibrinolysis in older people are justifiable. Advanced age is a clear predictor of major bleeding after fibrinolysis. In the STREAM trial, an excess rate of intracranial hemorrhage (ICH) in participants ≥75 years prompted a protocol amendment recommending a 50% decrease in the tenecteplase dose in this age group.^1^An extensive analysis of patients treated with full tenecteplase dose or alteplase has shown that the risk of major bleeding or ICH increases from ~60 years.^6^

Second, it is widely accepted that the benefits of coronary reperfusion with thrombolysis surpass the bleeding risk even in those individuals >75 years. Indeed, older age is not even a relative contraindication for fibrinolysis in STEMI.^3 , 4^In the post-protocol amendment phase of the STREAM trial, there was no ICH with half-dose tenecteplase, and the efficacy of the pharmacoinvasive strategy was maintained among 93 elderly participants.^7^

Third, clinicians must pay attention to particularities of the pharmacoinvasive treatment in older STEMI patients. Not only should the tenecteplase dose be cut in half in individuals ≥75 years, but the loading dose of 300 mg clopidogrel and the initial 30 mg intravenous bolus of enoxaparin should also be omitted. A 25% reduction in the enoxaparin dose is also recommended, at least in the initial phase of treatment. In addition, in the presence of renal dysfunction, the enoxaparin dose should be adjusted, or unfractionated heparin should be used.^3 , 4^Dosing errors of antithrombotics in older people with MI may be common and impose a higher risk of major bleeding.^8^

Fourth, the optimal antithrombotic regimen in older individuals in the context of a pharmacoinvasive strategy in STEMI is an evolving issue, and recommendations may change as new evidence emerges. For instance, the ongoing STREAM-2 trial evaluates the efficacy and safety of half-dose tenecteplase and antiplatelet therapy, including a loading dose of 300 mg clopidogrel, compared to standard primary PCI, in STEMI patients aged ≥60 years.^6^

The discussion on the safety of the pharmacoinvasive strategy should be put into the broader context of STEMI care quality. In Brazil, there is room for improvement in several aspects of STEMI treatment, including the rates of reperfusion therapy.^9^In the Brazilian Registry of Acute Coronary Syndromes (ACCEPT, patients included from 2010 to 2014), nearly 20% of the participants did not receive reperfusion therapy for STEMI, and this rate rises to ~35% in the Midwest and Northern Regions.^10^Late arrival at the emergency room and contraindications for fibrinolysis may account for several cases. However, some older individuals may not be prescribed fibrinolytic drugs due to concerns about the bleeding risk, which is not supported by the literature. In conclusion, older individuals constitute a special group of STEMI patients. Studies addressing the particularities of treatment in this population are welcome and necessary to provide adequate evidence to optimize medical care.
